# A comparative study of mNGS and tNGS for pathogen detection in paired upper and lower respiratory tract specimens from pediatric pneumonia patients

**DOI:** 10.1128/spectrum.01165-26

**Published:** 2026-09-24

**Authors:** Jinglin Zhao, Liping Zhang, Weizhi He, Xiaoli Jian, Dingrui Han, Adong Shen, Li Jiang, Xiaomei Liu

**Affiliations:** 1 Kunming Children’s Hospital (Kunming Medical University Affiliated), Kunming, Yunnan, People's Republic of China; Central Texas Veterans Health Care System, Temple, Texas, USA

**Keywords:** metagenomic next-generation sequencing (mNGS), targeted next-generation sequencing (tNGS), pediatrics, infectious diseases

## Abstract

High-throughput sequencing technologies have become essential tools in clinical diagnostics, but the comparative performance of targeted next-generation sequencing (tNGS) versus metagenomic next-generation sequencing (mNGS) in pediatric respiratory infections remains unclear. In this study, paired throat swab and bronchoalveolar lavage fluid (BALF) specimens were collected from children with lower respiratory tract infections. Both tNGS and mNGS were used to detect pathogens in paired samples. For the same specimen type, tNGS and mNGS showed strong agreement, with Kappa coefficients of 0.791 (*P* < 0.001) for BALF and 0.757 (*P* < 0.001) for throat swabs. Although the pathogen profiles of the upper and lower respiratory tracts differed, when using the same detection method, the agreement between the two anatomical sites remained substantial, with Kappa values of 0.659 (*P* < 0.001) for mNGS and 0.590 (*P* < 0.001) for tNGS—both approaching or reaching the threshold for strong consistency. These findings confirm that tNGS is a reliable alternative to mNGS for pediatric respiratory pathogen detection, and that even between different respiratory sites, the diagnostic agreement is high enough to support clinical decision-making.

**IMPORTANCE:**

This study demonstrates that, for the same specimen type, tNGS and mNGS achieve substantial agreement in detecting respiratory pathogens in children (Kappa 0.791 for BALF and 0.757 for throat swabs). Thus, tNGS offers a cost-effective alternative to mNGS for routine pediatric respiratory diagnostics. Importantly, despite distinct pathogen profiles between the upper respiratory tract (URT) and lower respiratory tract (LRT), substantial diagnostic concordance was observed between paired throat swab and BALF specimens tested with identical sequencing approaches (Kappa = 0.659 for mNGS, 0.590 for tNGS). This observation bears meaningful clinical relevance, suggesting that URT throat swabs could provide diagnostically helpful etiological clues. Still, BALF sampling retains critical value for identifying pathogens predominantly residing in the lower respiratory tract, and throat swab results require prudent clinical interpretation.

## INTRODUCTION

Respiratory infections have long posed a major global public health challenge, imposing a considerable medical burden that exceeds that of cancer and cardiovascular diseases (1, 2). A wide range of pathogens can cause respiratory illnesses, often resulting in both single and co-infections that lead to complex respiratory conditions and substantial risks to patient safety (3). In children, the clinical outcomes of respiratory infections are generally more severe than in adults. According to the Global Burden of Disease (GBD) 2021 study, pneumococcal infections alone caused approximately 179,354 deaths among children under five globally in 2021 (4). Furthermore, the World Health Organization (WHO) reported that pneumonia and diarrhea collectively accounted for an estimated 1.17 million deaths in children under five in 2021, representing 23% of all childhood deaths in that age group (5, 6). Respiratory tract infections in children predominantly occur during autumn and winter, typically exhibiting a relatively stable pattern of epidemic pathogens (7). However, notable shifts in the epidemiological characteristics of pediatric respiratory viral infections have been observed after the COVID-19 pandemic (8), including changes in the timing of epidemic seasons and in the diversity of circulating pathogens (9–11). These developments underscore the urgent need for rapid and accurate diagnostic tools.

Next-generation sequencing (NGS) has emerged as a transformative technology that enables comprehensive identification of infectious pathogens without prior culture. Compared to traditional culture-based methods, NGS offers higher sensitivity and aligns with the principles of precision medicine (12, 13). Growing evidence supports the superiority of NGS over traditional techniques. For instance, mNGS has been shown to outperform standard culture methods, providing greater sensitivity and reduced interference from prior antibiotic administration (14). Additionally, mNGS enabled the first detection of *Streptococcus canis* in the plasma of a patient with severe sepsis (15). The diagnostic utility of mNGS in infectious diseases has been extensively documented (16, 17).

More recently, tNGS has emerged in China as a promising alternative to mNGS. While mNGS employs a “shotgun” approach to unbiasedly sequence all nucleic acids in a sample, tNGS enriches and sequences only pre-specified target genes of pathogens using multiplex PCR or probe capture technology (18). tNGS offers the potential advantages of lower cost and higher sensitivity for specific targets, but its comparative performance in pediatric respiratory infections remains to be fully elucidated.

Therefore, this study was designed to compare the pathogen detection capabilities of mNGS and tNGS using the high-throughput sequencing platform at Kunming Children’s Hospital. By evaluating their discrepancies and concordance, we aim to shed light on optimal method selection, support clinical decision-making, and facilitate precision diagnosis for pediatric respiratory diseases.

## MATERIALS AND METHODS

### Sample collection

**Fig 1:**
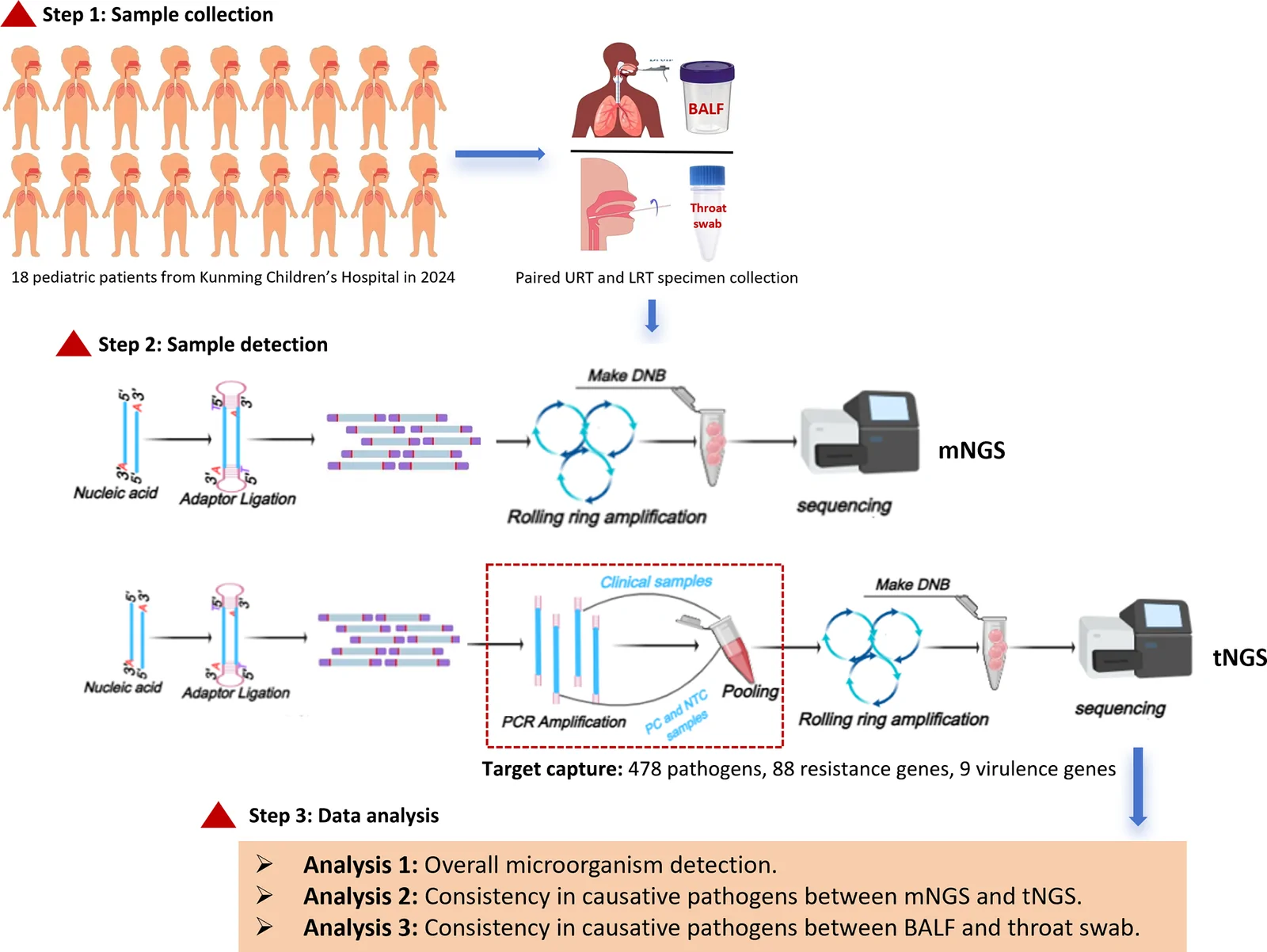
Study design and workflow of the whole experiment.

Paired BALF and throat swab specimens were collected from 18 pediatric patients admitted to the Department of Respiratory Medicine at Kunming Children’s Hospital in 2023, which has around 1,500 beds and 2 million admissions per year. For each participant, one throat swab and one BALF sample were collected for subsequent mNGS and tNGS analyses (Fig. 1). Clinical information from patients, including age, gender, medication use, and immune status, was transferred from medical records (seen in Table 1). The research was conducted in strict compliance with the ethical principles outlined in the Declaration of Helsinki and received approval from the Clinical Trial Ethics Committee of Kunming Children’s Hospital (approval number: 2024-03-073-K01). The specific inclusion and exclusion criteria are as follows.

**TABLE 1:** Detailed information and characteristics of 18 patients

Specimen	Gender	Age (Year)	Prior antibiotic use (Yes/No)	Immune status (corticosteroid therapy) (Yes/No)	Respiratory support (Yes/No)	Clinical diagnosis	Culture/microscopic examination	Molecular detection
Patient no. 1	F	4	No	Yes	Yes	Lobar pneumonia	Negative/negative	*Mycoplasma pneumoniae*
Patient no. 2	M	0.6	No	Yes	Yes	Lobar pneumonia	Negative/gram-positive cocci	Negative
Patient no. 3	F	3.2	Yes	Yes	Yes	Lobar pneumonia	Negative/–	Negative
Patient no. 4	F	6	Yes	Yes	Yes	Lobar pneumonia	Negative/negative	*Mycoplasma pneumoniae*
Patient no. 5	M	2.2	No	Yes	Yes	Lobar pneumonia	Negative/–	Negative
Patient no. 6	M	6	No	Yes	Yes	Lobar pneumonia	Negative/–	*Mycoplasma pneumoniae*
Patient no. 7	M	13	Yes	No	No	Lobar pneumonia	Negative/gram-positive cocci, gram-negative diplococci, gram-negative bacilli	Negative
Patient no. 8	M	1.5	Yes	Yes	Yes	Bronchopneumonia	Negative/negative	Negative
Patient no. 9	M	7	Yes	Yes	No	Bronchopneumonia	Negative/–	Negative
Patient no. 10	M	8	Yes	Yes	Yes	Lobar pneumonia	Negative/negative	*Mycoplasma pneumoniae*
Patient no. 11	F	3.1	Yes	Yes	Yes	Lobar pneumonia	Negative/gram-positive cocci	Negative
Patient no. 12	M	8	Yes	Yes	Yes	Lobar pneumonia	Negative/negative	*Mycoplasma pneumoniae*
Patient no. 13	M	12	No	Yes	Yes	Bronchopneumonia	Negative/gram-positive cocci	*Mycoplasma pneumoniae*
Patient no. 14	M	10	Yes	Yes	Yes	Lobar pneumonia	Staphylococcus aureus/gram-positive cocci	*Mycoplasma pneumoniae*
Patient no. 15	F	7	Yes	Yes	Yes	Lobar pneumonia	Negative/negative	*Mycoplasma pneumoniae*
Patient no. 16	M	6	Yes	Yes	Yes	Lobar pneumonia	Negative/–	*Mycoplasma pneumoniae*
Patient no. 17	F	8	Yes	Yes	Yes	Lobar pneumonia	Negative/negative	*Mycoplasma pneumoniae*
Patient no. 18	M	9	Yes	Yes	Yes	Lobar pneumonia	Negative/–	Negative

#### Inclusion criteria

1. Clinical diagnosis consistent with LRTs pneumonia: According to the Infectious Diseases Society of America (IDSA)/American Thoracic Society (ATS) guidelines, at least two of the following clinical manifestations should be present, along with imaging evidence of newly developed or progressive pulmonary infiltrates, consolidation, or ground-glass opacities:
  - Fever (temperature ≥38.0°C) or hypothermia (<36.0°C)
  - Newly developed cough, sputum production, or worsening of pre-existing respiratory symptoms.
  - Pulmonary consolidation signs and/or moist rales.
  - Peripheral blood leukocytes >10 × 10^9^/L or <4 × 10^9^/L, with or without left shift.
  - Respiratory failure or hypoxemia without other clear causes.
2. Planned bronchoscopy: The patient is scheduled to undergo bronchoscopy and collect BALF due to clinical diagnostic and treatment needs (such as failure of empirical treatment, suspected special pathogens, immunocompromised host, and severe pneumonia).
3. Qualified specimen: BALF recovery rate ≥30%, with no large number of squamous epithelial cells observed under the microscope to ensure alveolar origin.
4. Age: Age ≥1 month and <18 years.
5. Informed consent: The legal representative signs the informed consent form, agreeing to collect the remaining BALF specimens for both mNGS and tNGS testing.

#### Exclusion criteria

1. Absolute or relative contraindications for bronchoscopy and lavage, including severe coagulation dysfunction: PLT <50 × 10⁹/L or international normalized ratio (INR) >2.0.
2. Used new systemic antibiotics/antifungal/antiviral drugs within 48 h before sampling.
3. BALF sample quality is substandard.

### Sample processing and host cell removal

For each specimen, cell counting was performed using a cell counter following mixing with an equal volume of dye. Sputum and bronchoalveolar lavage fluid (BALF) samples were diluted threefold with phosphate-buffered saline (PBS) prior to counting. Host cell removal was performed based on total cell concentrations. Samples with a concentration below 1 × 10^6^ cells/mL were directly subjected to cell wall disruption at 18 m/s for 5 min. For samples exceeding 1 × 10⁶ cells/mL, centrifugation (14,000 × *g*, 3 min) was applied, followed by supernatant removal and resuspension of the pellet in PBS. The resuspended sample was then combined with a host removal reagent mixture, incubated at 37 °C with rotation (1,000 rpm, 10 minutes), washed with PBS, and finally subjected to cell wall disruption at 18 m/s for 140 s.

### Nucleic acid extraction and purification

DNA and RNA were extracted using a fully automated system with a prepackaged extraction kit (GensKey #MB2601001). Reagents were dispensed into designated wells, including lysis buffer, internal control, magnetic beads, wash buffers, and elution buffer. For RNA extraction, samples were diluted with nuclease-free water prior to loading.

### Library preparation and quantification

Library construction involved genomic DNA digestion, single- and double-stranded DNA synthesis, purification, fragmentation, end repair, and adapter ligation. For tNGS, there is a target amplify step when constructing the library. In the present study, a tNGS kit (GenSkey, Cat. no. P062601024) was applied for pathogen detection. This multiplex PCR-based tNGS panel covers a total of 475 pathogenic microorganisms, 88 antimicrobial resistance genes, 150 gene variants, and nine high-virulence genes. The complete target list of this tNGS panel is summarized in Table S1 of the supplemental materials. Notably, all pathogens identified by untargeted mNGS in our study were also within the detection scope of this tNGS kit. Final library concentrations were measured using a Qubit fluorometer with the dsDNA High Sensitivity assay.

### Library pooling and DNB preparation

For library pooling, 5 µL of each library was denatured (96°C, 2 min) and immediately cooled on ice. An aliquot of the denatured library was mixed with detection working solution, incubated in the dark, and quantified using the Qubit dsDNA High Sensitivity assay to guide pooling. DNA nanoball (DNB) preparation was performed on a rolling circle amplification instrument. After adding DNB termination buffer, the mixture was gently mixed. DNB concentration was assessed using a Qubit fluorometer with standards, and sequencing was initiated only for samples meeting quality thresholds (concentration >8  ng/µL).

### Pathogen identification

For pathogen identification, sequencing data quality control consists of two steps: wet-lab QC and bioinformatics QC. In wet-lab QC, positive and negative controls must pass, library concentration must be >1 µg/mL, and DNB concentration must be >8 µg/mL. In bioinformatics QC, Q30 must be >85%. After QC, candidate pathogens are preliminarily screened using the automated platform (Gensky) based on predefined reporting rules. Then, clinical microbiology experts further assess pathogen characteristics and experimental conditions. Finally, two experienced respiratory physicians determine the final report based on clinical symptoms and medication history.

### Statistical analysis

Statistical analyses were performed using SPSS software version 21.0 (https://www.ibm.com/support/pages/node/417815). The agreement between mNGS and tNGS for pathogen identification in clinical specimens was assessed using Kappa statistics. Additionally, Kappa analysis was utilized to evaluate the concordance of pathogen detection in specimens obtained from both the upper and lower respiratory tracts when analyzed with the same detection technique. According to Landis and Koch’s study (19), Kappa values were interpreted as follows: values below 0.2 indicated poor consistency; values between 0.2 and 0.4 denoted fair consistency; values from 0.4 to 0.6 represented moderate consistency; values between 0.6 and 0.8 reflected substantial consistency; and values ranging from 0.8 to 1.0 signified very strong consistency. Statistical significance was defined as a *P*-value < 0.05.

## RESULTS

### Clinical characteristics of the patients

The characteristics of the 18 patients were summarized at Table 1. The median age of the 18 patients was 6.5 years (interquartile range [IQR]: 3.2–8.3 years). Most patients were female (66.7%). Before NGS testing, 94.4% had received corticosteroid therapy, and 72.2% had received empirical antibiotic treatment. As we see, of the 18 patients in this cohort, 15 received a clinical diagnosis of lobar pneumonia, whereas three were diagnosed with bronchopneumonia.

### Pathogenic microorganisms’ distribution in low and upper respiratory tract samples detected by mNGS or tNGS

**Fig 2:**
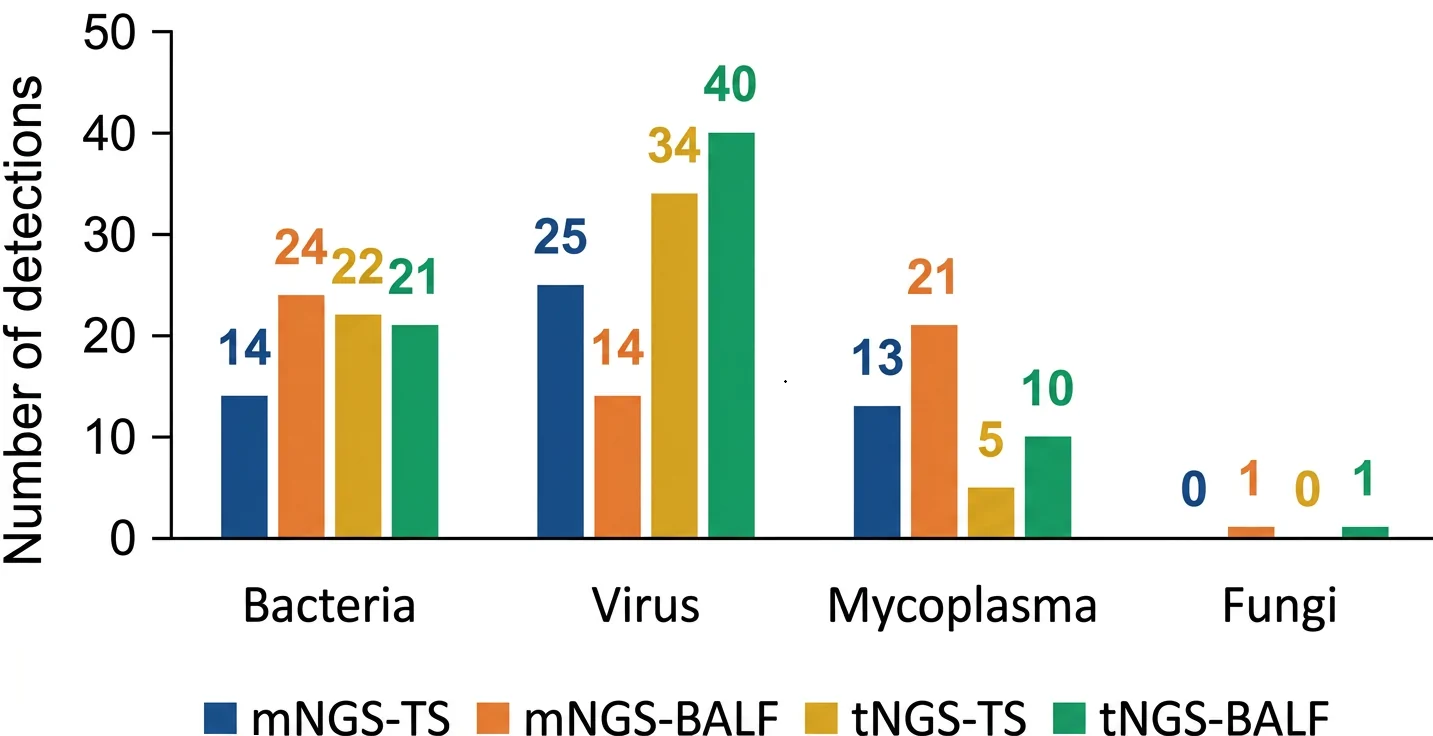
Performance of mNGS and tNGS in identifying causative pathogens in both BALF and throat swab.

As shown in Fig. 2, the application of mNGS and tNGS to BALF and throat swab specimens yielded 81 bacterial detections and 113 viral detections, with viruses outnumbering bacteria. *Mycoplasma* was identified 49 times, while fungi were detected only twice. All 18 samples tested positive for more than one pathogen (see File S2 for details).

**Fig 3:**
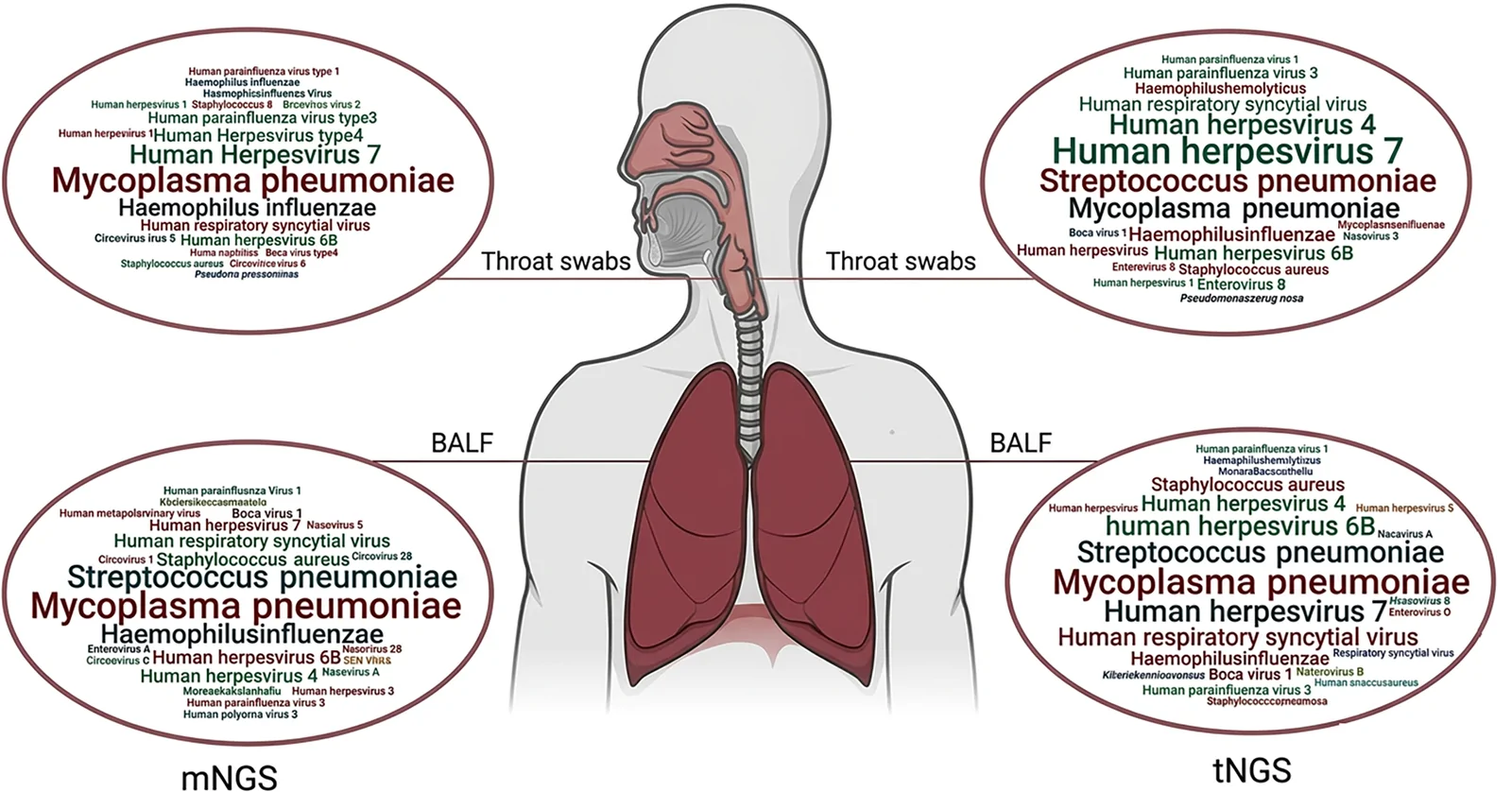
The Atlas of Pediatric Respiratory Pathogens at Kunming Children’s Hospital. Detected by mNGS and tNGS.

Additional species-level analysis is presented in Fig. 3. In BALF, mNGS identified 24 pathogenic microorganisms, with *Mycoplasma pneumoniae*, *Streptococcus pneumoniae*, and *Haemophilus influenzae* being the three most frequent. tNGS detected 23 pathogens in BALF, with the top three being *M. pneumoniae*, *S. pneumoniae*, and Human herpesvirus 7 (HHV-7). In throat swabs, mNGS detected 20 pathogens, predominantly *M. pneumoniae*, HHV-7, and *H. influenzae*; tNGS detected 19 pathogens, with *M. pneumoniae*, HHV-7, and *S. pneumoniae* showing the highest detection rates (Fig. 3).

### Diagnosis consistency in pathogenic microorganisms by mNGS and tNGS

As shown in Fig. 3, mNGS detected more pathogens than tNGS in both BALF and throat swab specimens. We then assessed the diagnostic concordance between the two methods.

**Fig 4:**
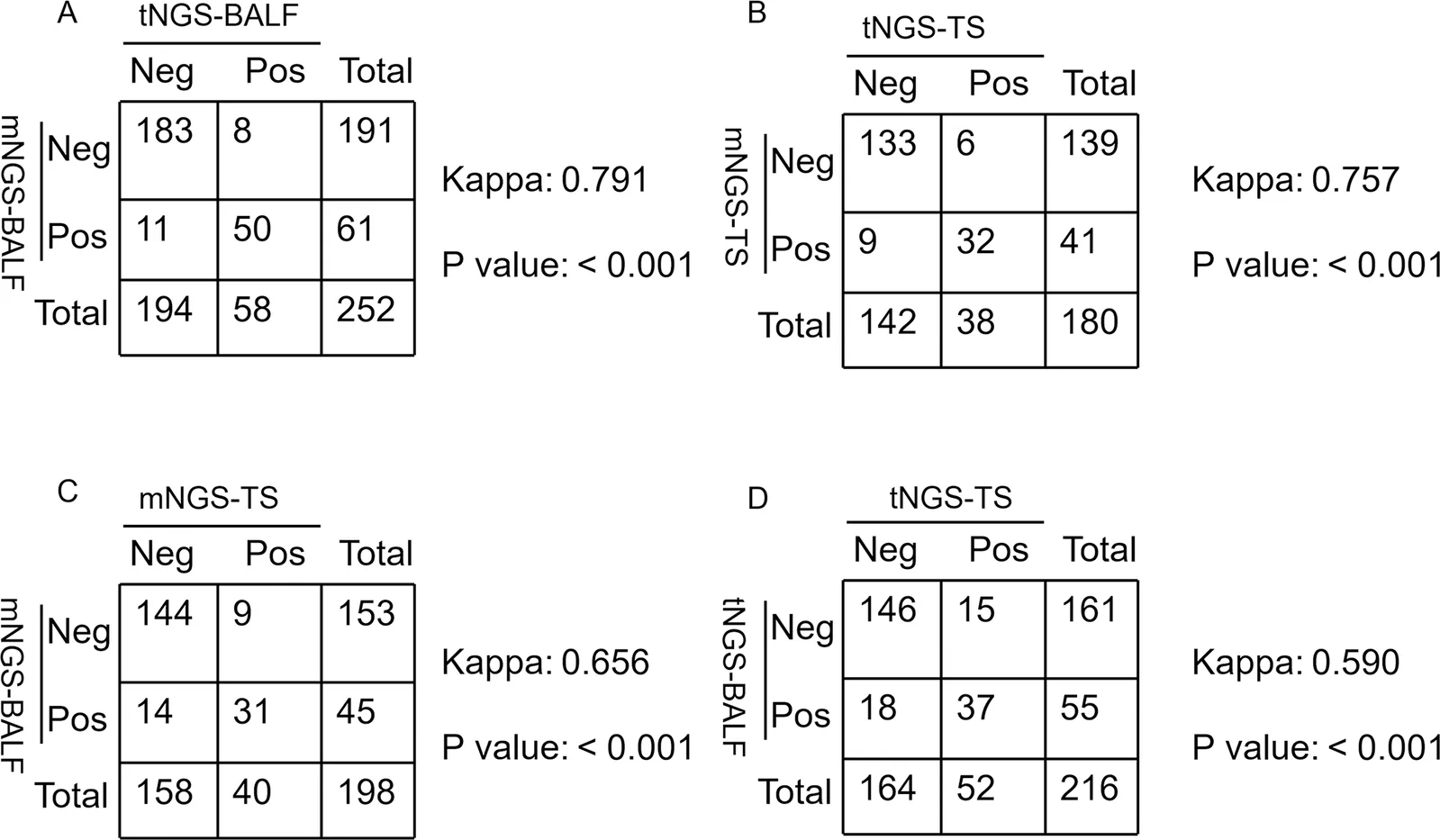
Pathogen detection consistency. (**A and B**) tNGS vs mNGS in BALF and TS; (**C and D**) BALF vs TS by mNGS and tNGS. Neg, negative; Pos, positive.

For BALF samples, substantial agreement was observed for multiple viruses (e.g., *Parvovirus*, *Human herpesvirus*, *Respiratory syncytial virus*, *Human parainfluenza virus*, and *Rhinovirus*) and bacteria (e.g., *Moraxella catarrhalis*, *Klebsiella pneumoniae*, and *Mycoplasma pneumoniae*) (Table 2). Overall, mNGS and tNGS showed substantial concordance in BALF (Kappa = 0.791, *P* < 0.001; Fig. 4A).

**TABLE 2:** Detailed consistency detecting pathogen information between mNGS and tNGS

	BALF mNGS vs BALF tNGS			Throat swab mNGS vs throat swab tNGS		
	Pathogen	Kappa	*P-*value	Pathogen	Kappa	*P-*value
Bacteria	*Klebsiella pneumoniae*	1	0.001	*Klebsiella pneumoniae*	1	0.001
	*Moraxella catarrhalis*	1	0.001	*Staphylococcus aureus*	0.64	0.004
	*Mycoplasma pneumoniae*	0.886	0.001	*Haemophilus influenzae*	0.571	0.007
	*Staphylococcus aureus*	0.679	0.004	*Mycoplasma pneumoniae*	0.4706	0.036
	*Streptococcus pneumoniae*	0.667	0.001			
	*Haemophilus influenzae*	0.336	0.093			
				*Human respiratory syncytial virus*	1	0
Virus	*Rhinovirus*	1	0.001	*Human parainfluenza virus*	1	0
	*Human polyomavirus*	1	0.001	*Enterovirus*	0.769	0.001
	*Human respiratory syncytial virus*	0.727	0.001	*Bocavirus*	0.64	0.004
	*Parvovirus*	0.609	0.005	*Rhinovirus*	0.64	0.004
	*Human herpesvirus*	0.556	0.016	*Human herpesvirus*	0.571	0.007

Similarly, in throat swabs, significant viral concordance was found for *Bocavirus*, *Human herpesvirus*, *Respiratory syncytial virus*, *Human parainfluenza virus*, and *Enterovirus*. Bacterial concordance was also high for *Staphylococcus aureus*, *Haemophilus influenzae*, and *K. pneumoniae* (Table 2). The overall agreement in throat swabs was substantial (Kappa = 0.757, *P* < 0.001; Fig. 4B).

### Diagnosis consistency in paired respiratory tract samples detected by mNGS and tNGS

To assess concordance between upper and lower respiratory tract specimens, paired BALF and throat swabs from the same patients were tested by both mNGS and tNGS.

Using mNGS, more pathogens were detected in BALF than in throat swabs. *Mycoplasma pneumoniae* and *Haemophilus influenzae* were the most common in both specimen types. Viral concordance was substantial for most viruses (e.g., *Respiratory syncytial virus*, *Parainfluenza virus*, *Enterovirus*), except for human herpesvirus which showed poor agreement. Bacterial concordance was high for *Klebsiella pneumoniae*, *M. pneumoniae*, and *H. influenzae* (Table 3). Overall, agreement between BALF and throat swabs was substantial (Kappa = 0.659, *P* < 0.001; Fig. 4C), indicating that despite differences in pathogen profiles, the two specimen types yield comparable diagnostic information.

**TABLE 3:** Detailed consistency detecting pathogen information between BALF and TS

	mNGS BALF vs mNGS throat swab			tNGS BALF vs tNGS throat swab		
	Pathogen	Kappa	*P-*value	Pathogen	Kappa	*P-*value
Bacteria	*Klebsiella pneumoniae*	1	0.001	*Klebsiella pneumoniae*	1	0.001
	*Mycoplasma pneumoniae*	0.886	0.001	*Pseudomonas aeruginosa*	1	0.001
	*Haemophilus influenzae*	0.64	0.006	*Haemophilus influenzae*	0.824	0.001
				*Streptococcus pneumoniae*	0.417	0.019
				*Mycoplasma pneumoniae*	0.259	0.2
				*Staphylococcus aureus*	0.217	0.32
					
Virus	*Enterovirus*	1	0.001	*Bocavirus*	0.769	0.001
	*Human polyomavirus*	1	0.001	*Human parainfluenza virus*	0.768	0.001
	*Human respiratory syncytial virus*	0.824	0.001	*Enterovirus*	0.769	0.001
	*Human parainfluenza virus*	0.769	0.001	*Human respiratory syncytial virus*	0.571	0.007
	*Bocavirus*	0.455	0.021	*Human herpesvirus*	0.271	0.28
	*Human herpesvirus*	−0.222	0.316			

With tNGS, pathogen detection was also higher in BALF than in throat swabs; *M. pneumoniae* and *Streptococcus pneumoniae* were the most common. Viral concordance was strong for *Bocavirus*, respiratory syncytial virus, parainfluenza virus, and enterovirus. Bacterial concordance was high for *H. influenzae*, *K. pneumoniae*, and *Pseudomonas aeruginosa* (Table 3). Similarly, overall agreement between BALF and throat swabs was substantial (Kappa = 0.59, *P* < 0.001; Fig. 4D), further confirming that upper respiratory specimens can serve as a useful proxy for lower respiratory tract pathogens in many cases.

## DISCUSSION

In recent years, high-throughput sequencing technology has advanced clinical pathogen identification and epidemiological investigations (20). Currently, mNGS and tNGS are the two main methods for pathogen detection; however, their diagnostic accuracy and clinical utility warrant further evaluation. In this study of 18 pediatric pneumonia patients with paired BALF and throat swab specimens, we observed substantial agreement between mNGS and tNGS in both BALF (Kappa = 0.791) and throat swabs (Kappa = 0.757), although tNGS detected slightly fewer pathogens overall, and agreement between upper and lower respiratory specimens was moderate (mNGS Kappa = 0.659, tNGS Kappa = 0.590).

Previous studies have reported that tNGS has comparable diagnostic efficacy to mNGS for lower respiratory tract infections, with advantages in fungal detection, and support its use as a cost-effective option (21). Another study found no significant differences in microbial detection rates between tNGS and mNGS across various specimen types (e.g., blood, BALF, sputum), with rates of 82.17% and 86.51%, respectively (22). These external findings align with our observation of substantial concordance between the two methods. Building on our findings and the technical advantages of tNGS, further discussion is warranted regarding the clinical application of these two methods in pathogen detection as well. In our cohort, tNGS detected slightly fewer pathogens overall compared to mNGS, but the difference was not clinically significant for most targeted pathogens, especially when common respiratory bacteria, viruses, and fungi were considered. This suggests that tNGS can reliably guide initial empirical therapy while reducing unnecessary broad-spectrum antibiotic use.

TNGS uses specific primers to enrich target pathogen genes, reducing sequencing data volume and improving sensitivity for selected targets while lowering costs, thus addressing limitations of mNGS such as high cost, low host-to-pathogen ratios of genomic DNA, and challenges in simultaneous DNA/RNA detection (23–26). This technological advantage supports the use of tNGS as a practical alternative for many clinical scenarios. A retrospective study of 190 cancer patients with suspected LRTIs demonstrated that tNGS achieved a significantly higher detection rate (91.6%) than CMC (24.2%), with superior sensitivity (96.1% vs 33.3%). Notably, 67.4% of patients tested positive exclusively via tNGS, and antibiotic therapy was modified for 41 patients based on tNGS findings, underscoring its clinical impact on treatment optimization (27). Our results demonstrated that mNGS detected 24 pathogens in BALF vs 23 by tNGS, and 20 vs 19 in throat swabs, indicating substantial concordance and suggesting that tNGS holds promise as a diagnostic useful tool for pediatric respiratory infections.

While respiratory tract infections are conventionally classified into upper and lower respiratory tract infections, recent epidemiological data have revealed increasing occurrences of dual-site co-infections and infections characterized by pathogen translocation between the upper and lower respiratory tracts (28–30). These clinical scenarios raise critical questions regarding whether non-invasive upper-respiratory-tract specimens can reliably represent lower-respiratory-tract pathogens. Co-infections are indeed prevalent in real-world clinical practice; a national survey conducted in China spanning 2009–2020 documented an overall co-infection rate of 23.3%, with the highest proportion (34.3%) observed among children (31, 32). Considering the invasive nature of BALF sampling in pediatric populations, we further evaluated the diagnostic concordance between throat swabs and BALF specimens. mNGS showed substantial agreement (Kappa = 0.659, *P* < 0.001), and tNGS also showed substantial agreement (Kappa = 0.59, *P* < 0.001). These Kappa values fall within or very close to the range for strong consistency (0.61–0.80). Therefore, despite inherent differences in pathogen distribution between the upper and lower respiratory tracts, the diagnostic yield from throat swabs is remarkably similar to that from BALF when analyzed by high-throughput sequencing. For certain pathogens (e.g., respiratory syncytial virus, influenza), throat swabs provide excellent concordance. For bacteria such as *Mycoplasma pneumoniae* and *Pseudomonas aeruginosa*, which preferentially colonize the lower tract, the agreement is still substantial, although negative results from throat swabs should be interpreted with caution. Taken together, our observations demonstrate a substantial level of diagnostic concordance between throat swabs and BALF detected by both mNGS and tNGS in this pediatric cohort. Higher agreement was achieved for pathogens such as respiratory syncytial virus and influenza virus, whereas moderate to substantial agreement was noted for LRTs predominant bacteria including *Mycoplasma pneumoniae* and *Pseudomonas aeruginosa*, for which negative throat swab results require prudent interpretation. These findings suggest that URT specimens may have potential utility as a less invasive sampling option for pediatric pneumonia, although this study alone cannot establish their generalizability as a formal clinical substitute for BALF. It should be emphasized that LRT sampling remains the gold-standard approach for definitive diagnosis, particularly in complex or severe pediatric cases.

High-throughput sequencing is increasingly integral to precision medicine (33). In the field of infectious disease diagnostics, both mNGS and tNGS have demonstrated significant clinical utility across various infection types (34). Our preliminary findings suggest that tNGS and mNGS have comparable diagnostic performance for most pediatric respiratory pathogens, enabling rapid and accurate diagnosis while reducing clinical burden. Also, our results indicate that throat swabs can provide referable etiological information for most pediatric respiratory infections and may serve as a promising minimally invasive sampling option in routine clinical scenarios, without completely replacing the gold-standard BALF sampling for severe or complicated cases. However, there are limitations. First, the small sample size (*n* = 18) limits generalizability; this study is exploratory. Second, it is a single-center study; results may not apply to other settings. Third, we did not perform culture or PCR on all samples, so true sensitivity could not be fully assessed. Fourth, the tNGS panel may not cover all emerging pathogens. Thus, larger, multi-center studies are needed to validate tNGS as a cost-effective alternative to mNGS. Extending tNGS to other specimen types (e.g, cerebrospinal fluid, blood) will help establish its broader diagnostic utility in pediatric infectious diseases.

### Conclusion

Our study confirmed that for the same specimen type, tNGS and mNGS achieve substantial agreement (Kappa = 0.791 and 0.757), supporting tNGS as a cost-effective alternative for pathogen detection. More importantly, despite distinct pathogen profiles between the URTs and LRTs, the diagnostic agreement between throat swabs and BALF was also substantial (Kappa = 0.659 for mNGS and 0.590 for tNGS), approaching the range of strong consistency. Collectively, these results indicate that upper respiratory specimens could yield informative and referable diagnostic data for most common pediatric respiratory pathogens, which may help reduce the reliance on invasive BALF sampling in clinical practice. In summary, tNGS exhibits favorable practicability and efficiency for routine pathogen screening, while mNGS retains unique value for the identification of unknown, atypical, or rare pathogens in pediatric respiratory infections.

## Data Availability

The original contributions presented in the study are included in the article/supplemental material; further inquiries can be directed to the corresponding author.

## SUPPLEMENTAL MATERIAL

File S1 spectrum.01165-26-s0001.xlsx Detailed information of target NGS panel.

File S2 spectrum.01165-26-s0002.xlsx Original sequencing data of mNGS in BALF samples.

ASM does not own the copyrights to Supplemental Material that may be linked to, or accessed through, an article. The authors have granted ASM a non-exclusive, world-wide license to publish the Supplemental Material files. Please contact the corresponding author directly for reuse.
